# Editorial: Microbial communities and functions contribute to plant performance under various stresses

**DOI:** 10.3389/fmicb.2022.992909

**Published:** 2022-11-07

**Authors:** Hui Li, Hai Ming Zhao, Diane Purchase, Xun Wen Chen

**Affiliations:** ^1^Department of Ecology, College of Life Science and Technology, Jinan University, Guangzhou, China; ^2^Department of Natural Sciences, School of Science and Technology, Middlesex University, London, United Kingdom

**Keywords:** microbiome, stress, function, crops, plant physiology

Plant-microbe interactions in natural and agricultural ecosystems have attracted more attention than ever (Toju et al., 2018; Berg and Cernava, 2022). In fact, these interactions are important to ecological functions and essentially relevant to the ecological services that humans rely on Fester et al. (2014). Harnessing the benefits of the interactions is one of the most considered and sustainable opportunities for further crop improvements (Bailey-Serres et al., 2019). Nevertheless, additional stresses due to anthropogenic activities are uncertain driving factors of ecological functions and services (Crutzen, 2002; Rudgers et al., 2020; Berg and Cernava, 2022). Climate change, agriculture-related stress, chemical pollution, and ozone depletion are typical environmental stresses in the Anthropocene (McGill et al., 2015; Dietz, 2017; Cavicchioli et al., 2019). These factors profoundly change the mode of plant-microbe interactions (Berg and Cernava, 2022). A better understanding of the influencing mechanisms and the ecological consequences are critical to maintaining sustainable natural and agricultural ecosystems.

Due to the extremely complex processes within the tripartite system (i.e., plant-microbiome-environment), harnessing repeatable and applicable benefits of microbes is definitely still in its infancy. Repeatability and deterministic processes could be observed under well-controlled experimental conditions, such as using a synthetic community (SynCom) of microbes (Niu et al., 2017; Liu et al., 2019; Zhang J. et al., 2021) to identify essential microbial mechanisms in facilitating plant performance, e.g., regulating suitable levels of phytohormone (Finkel et al., 2020), adjusting root endodermal permeability for nutrient acquisition (Salas-González et al., 2020), and resisting fungal pathogen by establishing a stable bacterial community (Niu et al., 2017). When it comes to the complex field condition, the coupled deterministic (e.g., environmental filters that select species with certain traits) and stochastic processes (chance events such as reproduction, death, and migration) (Zhou and Ning, 2017) and the influence of nonculturable microbial community (Lebeis, 2015) can often lead to difficulties in predicting the beneficial effects on plants.

Can plant microbiome really helps plants perform better in the way we desire, such as less disease and fertilizer with more yield? The question has been investigated from one-dimensional to multidimensional aspects: (1) symbiosis—symbionts of plants such as mycorrhizal fungi and rhizobia assist in nutrient acquisition and plant growth improvement (Smith and Read, 2008; Martin et al., 2017); (2) recruitment—protective microorganisms recruited by plants suppress the pathogen (Berendsen et al., 2012); (3) plant holobiont (an assemblage of species) and networking (i.e., microbe-microbe interactions)—assembly pattern determines plant health and fitness (Wei et al., 2015; van der Heijden and Hartmann, 2016; Niu et al., 2017; Finkel et al., 2020; Salas-González et al., 2020; Zhang L. et al., 2021). In the current Research Topic, Chen J. et al. further reviewed that harnessing the benefits of microbes is a promising approach to enhancing wheat performance under environmental stresses. Under such a backdrop, the present Research Topic collects studies investigating how microbes influence plant responses under typical stresses and their mechanisms, illustrating ongoing researches in this vivid and attractive field. The present Research Topic is categorized into sections considering different abiotic and biotic stress.

## Agricultural activity-related stress

Agricultural activities such as continuous cropping, fertilization, grazing, application of pesticides, use of plastic products (leading to micro and nanoplastic pollution), breeding, etc., are challenges for sustainable agriculture. The current Research Topic collects 24 papers that consider, among others, fertilization, continuous cropping, and grazing.

To improve crop yield, applying nitrogen (N) fertilizer in soils is an efficient approach. However, N input and overuse create N emission/leaching problems (Zhang et al., 2011) and drastically alter soil microbial activities (Chen et al., 2019). Nitrogen deposition due to air pollution is also an additional source of N input (Adams et al., 2021). In the current Research Topic, Gu et al. found that N application indeed changed bacterial diversity and community structures of sugarcane cropping systems. Excessive application of N fertilizers had unexpectedly led to a lower yield. This also echoes Chen J. et al.'s view that an optimized level of chemical input is needed to keep useful microbe functioning.

Apart from fertilization, continuous cropping is common in agriculture. Continuously planting the same or similar cultivars in the same soils often leads to plant growth inhibition and serious soil-borne diseases, which is known as continuous cropping obstacle (CCO) (Shipton, 1977; Xiong et al., 2015). The imbalance of soil microbiota with a reduced abundance of beneficial microbes has been considered one of the major reasons for CCO (Hiddink et al., 2010). Yuan et al. found that the rhizosphere of soybean suffered from CCO, showing an unstable rhizosphere microbial community. Yu J. et al. also found that CCO of garlic was related to more potential plant pathogens, fewer plant growth promoters, and compromised microbial diversity of soils. Chen D. et al. also observed similar alterations in microbial functions in the tobacco fields. Taken together, these results show a generalized phenomenon that continuous cropping brings chemical stress and leads to the instability of the microbial community. It provides further evidence that a stable microbial network is critical to maintaining plant fitness (Wei et al., 2015; van der Heijden and Hartmann, 2016; Niu et al., 2017; Finkel et al., 2020; Salas-González et al., 2020; Zhang L. et al., 2021). But we should note that the CCO is plant species dependent (Yuan et al.). For instance, Yang et al. reported that long-term (up to 30 years) alfalfa cultivation in the field enhanced soil microbial diversity and bacterial networking, higher than those short-term cultivations of meadows.

Overgrazing could lead to soil degradation, greenhouse gas emission, water pollution, and loss of biodiversity (Springmann et al., 2018; Wang et al., 2020). Grazing exclusion by fencing is one of the simplest and most common practices to restore degraded grassland. In addition to plant productivity and diversity, researchers started to look at the effects of grazing exclusion on soil microbial response. In the current Research Topic, Wang et al. show that the 4-year grazing exclusion did not increase the complexity and connectivity of bacterial co-occurrence patterns indicating a longer term of grazing exclusion is needed for soil restoration in terms of microbial activities. The optimal duration of grazing exclusion seems essential for the restoration depending on the type of ecosystem (Li et al., 2018; Song et al., 2020).

## Biotic stress

Plants can be infected by pathogens (i.e., bacteria, fungi, viruses, and nematodes) and attacked by herbivore pests (Atkinson and Urwin, 2012). The bacteria *Ralstonia solanacearum* can cause wilt disease in tobacco. Tan et al. investigated co-existence patterns of fungal communities in the rhizosphere and endosphere of tobacco infected by *R. solanacearum*. They found that the network structure was more complex under infection than under healthy conditions. The disease-resisting fungal genera may be suppressed due to the complex networking. It may let us re-think whether a complex microbial network is an indicator of a healthy plant holobiont.

The emission of volatile organic compounds (VOCs) triggered by plant growth-promoting rhizobacteria (PGPR) can resist herbivores. Herbivores can also induce plant volatile production. Understanding such chemical-mediated plant-insect interactions is important to achieve sustainable agriculture. Raglin et al. compared six maize genotypes with or without PGPR and herbivores, and they found that plant genotype was the main factor driving the levels and composition of VOC. Inoculation of PGPR did not influence VOC emissions but improved maize growth. It implies that plant genotypic variation is the dominant factor controlling the bacteria-mediated benefits. Although PGPR can improve plant growth, it is also critical to know how such an “alien” species/community affects the indigenous soil microbiota. Renoud et al. measured plant performance and functional groups in soils grown with maize in the fields and noted that inoculation of PGPR enhanced maize growth, and the functional groups were field-dependent.

## Drought and flooding

Plant-microbe interactions under drought stress are a popular research direction due to the widespread problem of the water crisis (de Vries et al., 2020). It is commonly reported that symbiotic fungi can alter soil water retention characteristics due to the production of glomalin (a glue-like substance) (Rillig and Mummey, 2006). In the current Research Topic, Cheng et al. review the roles of arbuscular mycorrhizal (AM) fungi (ancient and widespread symbiotic fungi of land plants) in helping their plant hosts to resist drought stress. Cheng et al. discuss the AM fungal diversity and activity, symbiotic relationship, morphological, physiological, and molecular mechanisms of AM fungi in assisting plant drought resistance. An outlook for future research is also provided. Not only fungi but also bacteria were considered to resist drought stress. Armanhi et al. study the impact of a synthetic community (SynCom) on the physiology and response of maize under drought stress. Results suggest SynCom inoculation reduced biomass loss and modulated vital physiological traits, such as lower leaf temperature, reduced turgor loss, and faster recovery upon rehydration. Gebauer et al. also determined the effect of water deficit history on soil microorganisms. They revealed interactive effects of soil type and water deficit condition. The approach allowed us to identify key microbial taxa promoting drought adaptation and improve the understanding of drought effects on plant-microbe interactions.

Different from the drought that leads to turgor pressure loss, embolism, and closure of stomata, precipitation and the subsequent flooding mostly increase the chance of leaf pathogen infection (Aung et al., 2018) and decrease rhizosphere oxygen levels hindering aerobic bacteria and mycorrhizal fungi (Unger et al., 2009). Francioli et al. found that flooding caused a significant reduction in wheat development and dramatic shifts in bacterial community composition at each plant growth stage, leading to detrimental effects on plant fitness and performance.

## Metal stress

Soil heavy metal pollution is widespread (Shi et al., 2018). Heavy metals accumulated in crops pose significant ecological and health risks (Zeng et al., 2019; Hu et al., 2020). Cadmium (Cd) species in soils is an essential factor in determining the risks. In the current Research Topic, Hao et al. investigated the vertical profiles of Cd in rice fields and found that soil pH, organic elements, and soil microbes are important drivers of Cd speciation. To mitigate Cd accumulation in rice, Kuang et al. applied lime and calcium-magnesium phosphate (CMP) amendments to paddy soils to reduce Cd bioavailability. The increased pH and phosphorus (P) in soils contributed to the decreased bioavailability of Cd and increased bacterial biodiversity. Also, Yu X. et al. found that using a Cd-immobilizing bacterial agent together with fermented organic matters in Cd-polluted soils could reduce plant (*Houttuynia cordata*) Cd uptake. Such amendments increased soil bacterial diversity.

Arsenic (As) pollution is also another classic and severe environmental problem (Huang et al., 2019; Li et al., 2021). Iron-oxidizing bacteria (FeOB) show the potential to mitigate As pollution, because FeOB could oxidize Fe(II) and provide As binding sites, which reduce As bioavailability (Emerson et al., 2010). In the current Research Topic, Qian et al. investigated the effect of FeOB inoculation on the As migration and transformation in paddy soils. They found that inoculation of *Ochrobactrum* sp. increased As proportion in the binding fraction. The reduced As bioavailability in soils led to less As uptake in rice tissues.

Copper (Cu) is another heavy metal of concern (Tani and Barrington, 2005a,b; de Vries et al., 2013). The alfalfa-rhizobium symbiosis can resist Cu stress, but the regulatory mechanism is unclear. In the current Research Topic, Duan et al. assessed the effects of rhizobium inoculation on the growth of alfalfa and soil microbial characteristics under Cu stress. They found that rhizobium inoculation markedly alleviated Cu-induced growth inhibition in alfalfa by increasing the chlorophyll content, height, and biomass, in addition to N and P contents. This study provides insights into the mechanism of action of the legume-rhizobium symbiotic system to mitigate Cu stress.

The stress of multiple metals presents a complex influence on the plant holobiont. Metal stress is commonly the critical factor inhibiting revegetation of mine tailings (Wong, 2003; Li, 2006). The tailing soil could be seriously poor in nutrients, which complicates the revegetation process. Liu et al. investigated the direct planting of *Pennisetum giganteum* into tailing sand with the designed bio-matrix pots, which were made by mixing corn cob powder and stalk powder. The *P. giganteum* could grow well, especially those in the bio-matrix pots, which had more nutrients and suitable microbial communities. However, in terms of ecological restoration, the key factors driving the recovery of ecological functions are not still clear. By integrating the factors of plant species, soil communities, and abiotic conditions, Zhu et al. provide a conceptual framework considering the plant-soil feedbacks (PSFs) (Bever, 1994; Rinella and Reinhart, 2018) to guide a better understanding of the mechanisms of the restoration process. Through this framework, we could enhance our ability to predict and optimize above- and below-ground communities for better restoring ecosystem functions.

## Salinity and habitats or cultivation type

Soil salinity is one of the most important abiotic factors limiting plant productivity (Bernstein, 1975; Zhao et al., 2020). Exposure to salts leads to osmotic and ionic stresses. Salt-tolerant endophytes could assist plants in salt tolerance through accumulating osmolytes, improving ion homeostasis and nutrient uptake, and providing phytohormones (Otlewska et al., 2020). In the current Research Topic, Szymańska et al. found that the isolated salt-tolerant endophytes could promote the growth of all tested plant species, suggesting a universal ability of endophytes to assist plant salt tolerance.

Since the soil microbial community affects the growth, quality, and yield of plants, understanding the microbial ecology of the agroecosystem could provide hints for sustainable agriculture. Kui et al. characterized the microbiome of tea plantation soils (448 soil samples from 101 ancient tea plantations). The authors revealed that the bacterial community was sensitive to environmental factors while the fungal community was more responsive to farmer intervention.

Among natural ecosystems, the mangrove ecosystems represent unique and essential habitats serving as sensitive areas for shoreline ecological functions (Lugo and Snedaker, 1974). Mai et al. found that different mangrove species harbor distinct microbial taxa in the sediments. Specific microbial taxa associated with the species *R. apiculata* contributed the highest functional activities related to carbon metabolism, carbon fixation, and methane metabolism. It indicates that mangrove-microbe interaction is species-dependent regarding the carbon cycle.

## Summary and prospects

The Research Topic themed on *Microbial communities and functions contribute to plant performance under various stresses* brings a diverse research collection to this key framework. The collection is not only targeting agricultural production but also involves ecological restoration and functions. The effects of microbes on plant performance and their mechanisms are complex, and they mostly seem site- and species-dependent, and comparability among studies could be improved. Combined stresses, rather than single stress, could be more typical in reality. Future studies shall focus on theories with more fundamental biological and ecological mechanisms for better generality leading to possible application.

## Author contributions

XWC and HMZ drafted and revised the manuscript. HL and DP commented on and edited the manuscript. All authors contributed substantially to the final version and approved it for publication.

## Funding

Supports from the National Key Technology R&D Program of China (2020YFC1807604), Guangdong Natural Science Fund for Distinguished Young Scholar (2021B1515020014), and the National Natural Science Foundation of China (42277211, 41877350, and 42077298) are gratefully acknowledged.

## Conflict of interest

The authors declare that the research was conducted in the absence of any commercial or financial relationships that could be construed as a potential conflict of interest.

## Publisher's note

All claims expressed in this article are solely those of the authors and do not necessarily represent those of their affiliated organizations, or those of the publisher, the editors and the reviewers. Any product that may be evaluated in this article, or claim that may be made by its manufacturer, is not guaranteed or endorsed by the publisher.
